# Predictive Factors of Better Patient Satisfaction After Phenol‐Croton Peel: A Retrospective Study of 102 Patients

**DOI:** 10.1111/jocd.70609

**Published:** 2026-01-25

**Authors:** Gustavo Carneiro Nogueira, Raquel Iracema de Freitas Macedo Oliveira, Mariana Rocha Andrade, Bárbara Arze Rocha, Naides Carneiro Nogueira, Márcio Roberto Silva, Ana Carolina Tardin Rodrigues de Medeiros, Marina Vieira Rodrigues de Queiroz, Felipe Xavier Clo, Ticiano Teixeira Cesar Clo, Carlos Gustavo Wambier, Gisele Viana de Oliveira

**Affiliations:** ^1^ Completa Dermatology Itaúna Minas Gerais Brazil; ^2^ Faculty of Medical Sciences of Minas Gerais (FCMMG) Belo Horizonte Minas Gerais Brazil; ^3^ EMBRAPA Dairy Cattle Juiz de Fora Minas Gerais Brazil; ^4^ Federal University of Juiz de Fora Juiz de Fora Minas Gerais Brazil; ^5^ Fibonaccy Plastic Surgery Belo Horizonte Minas Gerais Brazil; ^6^ The Warren Alpert Medical School of Brown University Providence Rhode Island USA; ^7^ Santa Casa de Misericórdia de Belo Horizonte Belo Horizonte Minas Gerais Brazil; ^8^ Luxemburgo Hospital Belo Horizonte Minas Gerais Brazil

**Keywords:** croton, phenol, rejuvenation

## Abstract

**Background:**

Phenol‐croton peels are the gold standard for treating sun‐damaged skin and static wrinkles; their long‐term outcomes and patient satisfaction rates have yet to be thoroughly investigated.

**Aims:**

To evaluate patient satisfaction and both short‐ and long‐term cutaneous side effects in individuals undergoing phenol‐croton peels.

**Materials and Methods:**

This retrospective cohort study analyzed 102 female patients who underwent phenol‐croton peels and were followed up until 3 months after the procedure to assess short‐term side effects and to identify any long‐term side effects that persisted beyond this period. Univariate and multivariate analyses were performed to assess patient satisfaction and long‐term cutaneous side effects.

**Results:**

Ninety‐two percent of patients rated their satisfaction as 4 or 5. Persistent cutaneous side effects were observed in 12% of cases (hypopigmentation: 6, hyperpigmentation: 5, dryness: 1). Despite mild hypopigmentation, five patients expressed willingness to undergo the procedure again. In the univariate analyses, full‐face treatment, increasing age, and the absence of cutaneous side effects were significantly associated with higher satisfaction scores (*p* < 0.05). In the multivariate model, age and the absence of cutaneous side effects remained independently and significantly associated with the outcome. Full‐face treatment, although not statistically significant in the final model, showed a trend toward association and contributed to the overall adjustment. Notably, cutaneous side effects decreased at follow‐up performed at least 15 months after the procedure compared to follow‐up conducted within 3 months post‐procedure (*p* = 0.039).

**Conclusions:**

Phenol‐croton peels demonstrated high satisfaction rates despite occasional prolonged cutaneous side effects. However, these side effects significantly decreased at ≥ 15 months post‐procedure compared to ≤ 3 months post‐procedure. Careful patient selection and expertise in performing this procedure remain crucial for optimizing outcomes.

## 1 Introduction

Phenol‐croton peelings are recommended for skin rejuvenation, having been used for several years by plastic surgeons and dermatologists. This procedure improves the appearance and texture of the skin by stimulating cell regeneration and reshaping [1, 2, 3, 4]. Phenol is a volatile solid with a characteristic odor, with almost instantaneous analgesic power, and croton oil is largely responsible for the depth achieved by the peeling [3, 4, 5, 6]. The Baker‐Gordon formula used a combination of Phenol and Croton oil [2] and it was the most used formula until studies demonstrated that the croton, not phenol, was the responsible ingredient for the depth of penetration and neocolagenesis [2, 4].

Phenol‐croton peeling is known to deliver effective results in a relatively short time, providing smoothing of wrinkles, skin rejuvenation, improvement of acne scars and melasma [7, 8], with a potential to improve not only appearance and self‐esteem, but also patient's quality of life [9]. However, most articles use a relatively small number of patients [6, 7, 10]. Although these are properly conducted studies, series with a larger number of patients submitted to phenol‐croton peels are necessary to assess overall patient's satisfaction, short‐ and long‐term cutaneous side effects and its rates.

There are several compounding formulas for phenol peel [4, 11, 12, 13, 14]. Baker‐Gordon has a combination of 2.1% croton oil and 49.3% phenol. Liquid soap with hexachlorophene and trichlosan is added as a surfactant. Dr. Hetter has suggested varying the concentrations of croton oil, while phenol is used at a fixed 35% concentration. He proposes a stock solution with 1 mL croton and 24 mL of 88% phenol, with a final stock solution concentration of 0.04 mL croton (1 drop). Hetter formulas have always a 35% phenol with croton concentrations varying from 0.2% to 1.6%. Formulas with 1.6% croton oil are used to treat thick, oily skin, while less concentrated croton oil peels are usually used in thin or very dry areas [4, 11, 12, 13, 14].

Phenol‐croton peels have lately gained popularity among Brazilian patients, and they have been performed regularly for our group, using the Hetter formulas. While phenol‐croton peels are usually associated with remarkable outcomes, cutaneous side effects sometimes occur, some of them persisting for several months [14, 15, 16]. We aimed in this study to evaluate the patient's satisfaction with the procedure and investigate the percentage of early (short‐term) and late (long‐term) side effects following the peel. Finally, all patients with long‐term cutaneous side effects were reinterviewed in June 2024.

## 2 Materials and Methods

### 2.1 Study Design, Setting, and Participants

This was a retrospective cohort study of 102 patients treated with phenol‐croton peeling carried out in an outpatient dermatology setting, evaluated according to the outcomes: global satisfaction index (GSI) and cutaneous side effects.

Exclusion criteria were patients less than 18 years, pregnant or breastfeeding, history of uncompensated cardiological disease, and patients with recent (< 1 month) or ongoing infection on the face, mainly herpes, and patients who underwent other facial ablative procedures (microneedling, laser resurfacing, or chemical peels). Participants included in this study were female individuals who underwent the full‐face (*n* = 25) or localized (*n* = 77) phenol‐croton peeling procedures, had a skin phototype ranging from I to III according to the Fitzpatrick classification, and underwent the peeling from 2015 to 2023.

The included participants were followed up in two stages. The first follow‐up occurred during the interview with all 102 patients, assessing both short‐term (up to 3 months) and longer‐lasting side effects (more than 3 months). The second follow‐up consisted of a second interview conducted in June 2024, exclusively with patients who had reported long‐term side effects, to reassess persistence of symptoms at ≥ 15 months post‐peeling.

This study was approved by the Ethical Committee (#5.943.455) and all patients provided written informed consent; participants did not receive financial compensation. Herein, we present findings from the analyses on short and long‐term side effects and patient's satisfaction. This study followed the Strengthening the Reporting of Observational Studies in Epidemiology (STROBE) guideline for cohort studies.

### 2.2 Data Collection

A structured questionnaire was used to interview the patients. A database of all individuals who had undergone phenol‐croton peeling between 2015 and 2023 was created. Interviewers, previously trained by a researcher experienced in structured interviews, conducted the interviews via pre‐scheduled telephone or digital calls in October and November 2023. Importantly, the interviewers did not participate in the clinical procedures to avoid embarrassment or bias in the responses. The study objectives were explained to each participant, who then digitally signed the informed consent form.

During the first round of interviews, all 102 patients who had undergone the procedure were contacted. Patients reported whether or not they experienced any side effects that persisted beyond 3 months following the procedure.

A second interview was conducted in June 2024, but only with the subset of patients (*n* = 12) who had reported persistent side effects in the first interview. This second round of interviews aimed to assess: (1) the persistence of the reported side effects; (2) overall satisfaction despite the adverse effects; and (3) whether patients would choose to undergo the procedure again knowing the outcomes. The cutaneous side effects surveyed included hyperchromia, hypochromia, dryness, herpes simplex, erythema, and scarring.

Patients who could not be reached due to changed phone numbers or having moved to other cities were considered lost to follow‐up.

Cutaneous side effects were classified as short‐term when they persisted for up to 3 months and long‐term when they lasted more than 3 months. The main exposures analyzed in this cohort study were the type of procedure (localized versus full‐face) and the time elapsed since the procedure. The primary outcomes were the GSI and the presence of cutaneous side effects. Appendix A summarizes the methodology of the study.

### 2.3 Phenol Formulas Used in This Study

The Hetter formulas were used in all patients, always with a 35% phenol with croton concentrations varying from 0.2% to 1.6%. Formulas with 1.6% croton oil were used to treat thick, oil skin, while less concentrated croton oil peels (0.2% to 0.4%) were performed in thin areas like the lower eyelids [9].

### 2.4 Data Analyses

Descriptive statistics on GSI and cutaneous side effects were shown. As this is a retrospective cohort study, in addition to descriptive statistics, univariate and multivariate linear regression models were used to evaluate associations with the GSI outcome. Similarly, chi‐square analysis was attempted to evaluate possible explanatory associations with the categorical side effects outcome. All variables with a *p* value < 0.20 in the univariate analysis were included in exploratory multivariate models, using the backward stepwise method to obtain the final model with the best fit.

Paired analysis to evaluate possible reduction of side effects was performed comparing side effects persisting for more than 3 months (long‐term) and those persisting for at least 15 months of follow‐up after the procedures, taking as reference the first follow‐up period (up to 3 months). The McNemar test was used to evaluate possible significant differences between the compared follow‐up periods.

## 3 Results

We were able to reach and interview 102 patients and all agreed to participate. A second follow‐up was conducted in June 2024 with the 12 patients who had reported long‐term side effects. 25 patients underwent the full‐face peel, and 77 received the localized peel (Table 1). Patients were all females with phototypes varying from I to III (Table 1). Patients' ages varied from 31 to 76 years, with a mean value of 57.3 years (SD = 9.30).

**TABLE 1:** Description of the study sample.

Variable	Frequency (%)
Treatment	
Fullface	25 (24.5)
Partial	77 (75.5)
Phototype	
1	27 (26.5)
2	55 (53.9)
3	20 (19.6)
Satisfaction	
Minimum	1.0
Maximum	5.0
Median	5.0
Mean	4.5
Standard deviation	0.84
Percentage ≤ 4.5	31.4
Percentage = 5.0	68.6
Side effects up to 3 months	
No	86 (84.3)
Yes	16 (15.7)
Side effects more than 3 months	
No	90 (82.2)
Yes	12 (11.8)
Side effects at least 15 months after the procedure	
No	95 (93.1)
Yes	7 (6.9)
Age	
Minimum	31
Maximum	76
Median	59.0
Mean	57.3
Standard deviation	9.3

**FIGURE 1:**
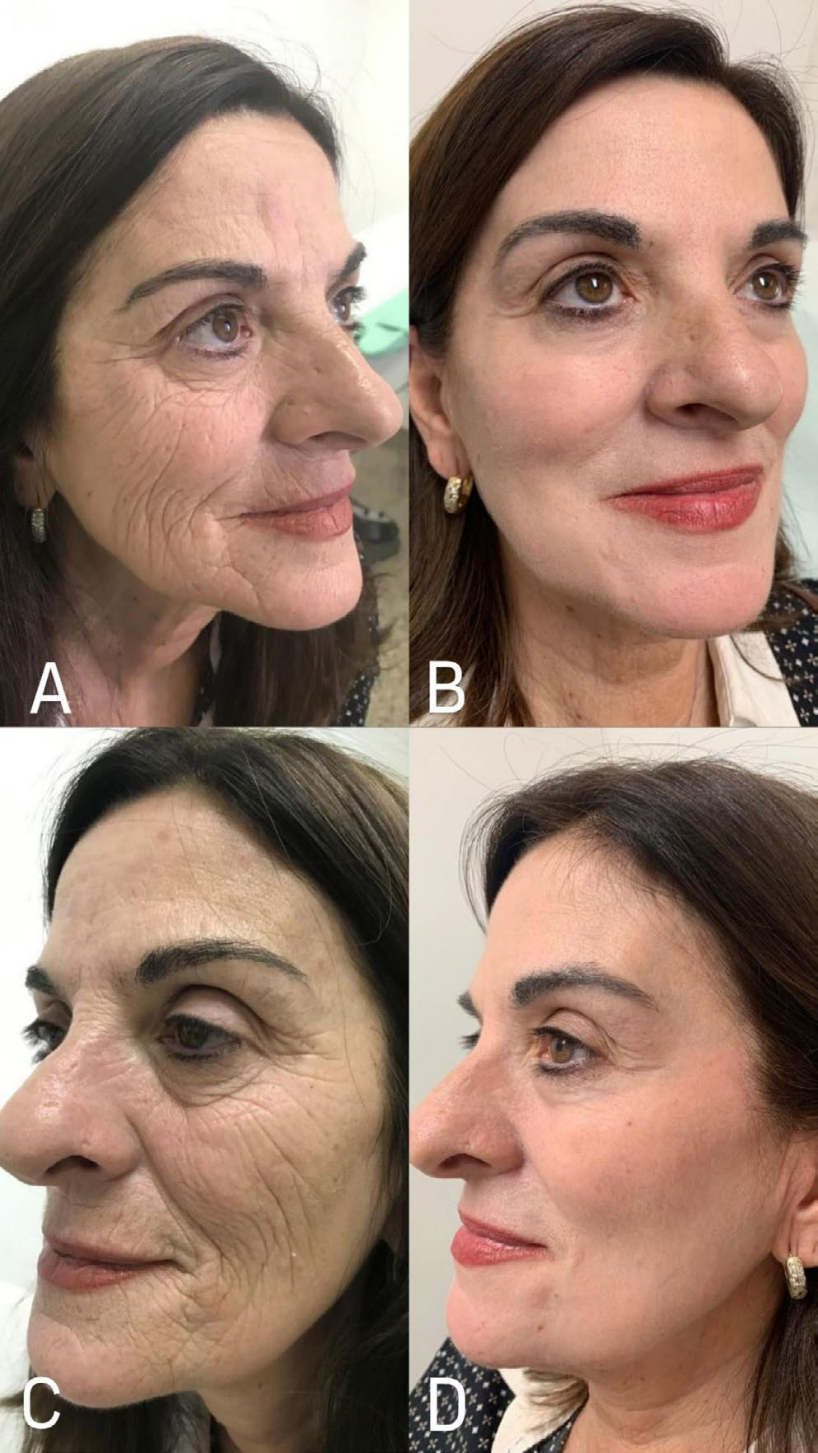
Pre‐treatment (A, C) and post‐treatment (B, D) photographs illustrating the outcome of phenol peeling, highlighting the enhanced skin surface and reduction of skin imperfections.

The majority of our patients were very satisfied, with 92% rating the procedure 4 and above (on a scale of 1 to 5); 70 of them (68.6%) self‐declared maximum satisfaction, rating the peel with a score of 5 (Table 1; Figure 1).

Regarding cutaneous side effects, 16 of the patients (15.7%) reported short‐term cutaneous side effects that persisted up to 3 months following the peeling, and 12 (11.8%) referred to cutaneous long‐term side effects that remained for more than 3 months (Table 1). Among the reports of short‐term cutaneous side effects, 7% of patients referred to some degree of hypopigmentation, while 6% referred to hyperpigmentation in treated areas. One patient reported dryness, one mentioned erythema, and one had a diagnosis of herpes simplex. Regarding the long‐term ones, six patients reported persistent hypochromia, five patients reported hyperchromia, one patient referred to cutaneous dryness, and none of the patients referred to a diagnosis of herpes simplex or persistent erythema.

In the second follow‐up, conducted in June 2024 with patients who had previously participated in the study, only one patient reported that she would not undergo the peeling procedure again. The adverse effects reported included hypochromia (6 patients) and hyperchromia (1 patient). Although some patients reported persistent hypopigmentation following the procedure, this was not perceived as a significant limitation to treatment satisfaction. While hypochromia can be considered a cutaneous side effect and potentially undesirable from the patient's perspective, it was not regarded as severe or disfiguring, such as a hypertrophic scar would be. On the contrary, even patients who experienced hypopigmentation expressed that the overall benefits of the treatment outweighed this effect, and most stated they would undergo the procedure again. These findings reinforce the idea that, although present, this type of side effect does not compromise the perceived efficacy and acceptability of the phenol‐croton peeling.

Correlations, univariate and multivariate analyses: The following variables, treatment type, age, cutaneous side effects up to 3 months (short term), those persisting for more than 3 months (long term), and those persisting for at least 15 months of follow‐up after the procedures, were associated with GSI in the univariate analysis.

However, the variables cutaneous side effects up to 3 months, more than 3 months, and at least 15 months post‐procedure showed moderate to high correlations with each other, raising concerns about multicollinearity. Thus, when these three variables were included simultaneously in a stepwise backward multivariate model—together with treatment type and age—only side effects more than 3 months, treatment type, and age remained in the final model, as they had the strongest explanatory power. Accordingly, this side effect variable also had the lowest *p* value in the univariate analysis.

Each additional year of patient age and the absence of cutaneous side effects were significantly associated with increases in GSI scores, by 0.018 and 0.96, respectively. Full‐face treatment was significantly associated with better GSI scores in the univariate analysis and showed a trend toward association in the multivariate model, suggesting a possible causal relationship that warrants further investigation, particularly in studies with larger sample sizes. Together, the three variables that remained in the final model accounted for an adjusted *R* ^2^ of 0.21 (Table 2). On the other hand, the chi‐square test did not find any of the variables tested associated with side effects (*p* > 0.05), so they were not tested in multivariate logistic regression models (Table 3).

**TABLE 2:** Univariate and multivariate linear regression analyses for global satisfaction index.

Variable	Univariate		Multivariate	
	Linear regression coefficient (*β*)	*p*	Linear regression coefficient (*β*)	*p*
Treatment				
Partial	Reference		Reference	
Fullface	0.48	0.012	0.30	0.084
Age (not categorical)	0.023	0.008	0.018	0.021
Phototype		0.08	—	—
1	Reference			
2	0.37	0.058		
3	−0.001	0.99		
Side effects up to 3 m				
No	0.73	0.0012	—	—
Yes	Reference			
Side effects more than 3 months				
No	1.05	0.000027	0.96	0.000068
Yes	Reference		Reference	
Side effects at least 15 months after the procedure				
No	0.82	0.012	—	—
Yes	Reference			

*Note:* Multivariate model summary: Adjusted *R* ^2^: 0.2186; *F*‐statistic: 10.42 on 3 and 98 df, *p* value: 5.195e‐06.

**TABLE 3:** Chi square analyses for side effects.

Variable	Total	Frequence (%)	*p*
Treatment			0.15
Partial	77	11 (14.3)	
Fullface	25	1 (4.0)	
Age (not categorical)	—	—	0.89
Phototype			0.84
1	27	4 (14.8)	
2	55	6 (10.9)	
3	20	2 (10.0)	

The proportion of cutaneous side effects showed a decreasing trend over the follow‐up periods after the procedure. Although this reduction was not statistically significant more than 3 months (4/16 or 25%, *p* = 0.12), it became significant beyond 15 months of follow‐up (9/16 or 56.25%, *p* = 0.0039), both in comparison to the first follow‐up (up to 3 months). Additionally, the reduction observed between the more than 3 month‐ and beyond 15‐month follow‐up periods was borderline significant (5/16 or 31.25%, *p* = 0.062) (Table 4).

**TABLE 4:** Paired analysis of reduction of side effects.

Total	Reference follow‐up	Comparison follow‐up	P‐P^a^	N‐N^a^	P‐N^a^	N‐P^a^	*p* ^b^
102	Up to 3 m	More than 3 m	12	86	4	0	0.12
102	Up to 3 m	At least 15 m	7	86	9	0	0.0039
102	More than 3 m	At least 15 m	7	90	5	0	0.062

^a^ P‐P: positive side effects in both follow‐up periods; N‐N: negative side effects in both follow‐up periods; P‐N: positive and negative side effects in reference and comparison follow‐ups, respectively; N‐P: negative and positive side effects in reference and comparison follow‐ups, respectively.

^b^ *p* value by the McNemar's test using exact binomial probability calculations.

## 4 Discussion

This study identified three key factors associated with higher GSI scores following phenol–croton oil peeling: full‐face treatment, older patient age, and the absence of cutaneous side effects. Patients undergoing full‐face application reported greater satisfaction, likely due to the more homogeneous aesthetic outcomes and improved facial harmony achieved when compared to segmented or localized treatments [22]. Additionally, older patients tended to express higher satisfaction, possibly because the rejuvenating effects are more perceptible in individuals with more advanced signs of aging, an association also noted in other facial aesthetic procedures [21]. Notably, the absence of complications such as hypopigmentation, hyperpigmentation, or dryness strongly correlated with higher GSI ratings, aligning with previous literature that highlights the negative impact of post‐procedure adverse events on patient satisfaction [20]. These findings underscore the importance of proper technique and patient selection to optimize outcomes in deep resurfacing procedures.

Phenol–croton oil peels are a well‐established modality for the treatment of wrinkles and chemical skin rejuvenation. Currently, no other resurfacing procedure offers such long‐lasting effects as phenol‐based peels. Previous histological studies have demonstrated durable improvements in dermal architecture, including sustained neocollagenesis observed 15–20 years after deep chemical peeling, with clear distinctions between treated and untreated photoaged skin areas [15].

In the present study, we observed other complementary results, such as a high rate of patient satisfaction and minimal reports of significant adverse effects. Notably, no cases of scarring were observed. However, seven patients reported hypopigmented areas within the first 3 months after the procedure. These findings should be interpreted with caution, as hypopigmentation is typically considered a late‐onset side effect following phenol peels. It is possible that some participants misinterpreted early post‐inflammatory changes, such as erythema or transient hyperpigmentation, as hypopigmentation [2]. This phenomenon has also been described in patients with higher phototypes who exhibited early post‐procedure depigmentation followed by gradual repigmentation [17].

Persistent hypopigmentation was reported by six patients in our cohort. This complication may result from deeper dermal injury or melanocyte dysfunction. Historically, it was more frequently associated with more aggressive formulations, such as the original Baker–Gordon peel [2]. Our team maintains long‐term follow‐up with all phenol‐treated patients. Among those with persistent hypopigmentation, only one patient reported continued dissatisfaction, specifically due to a small, visible area on the lower eyelid. The remaining five patients considered the hypopigmentation to be mild and not impactful on their overall treatment satisfaction. Despite the low incidence and subjective burden of this adverse effect, these findings highlight the importance of careful case selection and technical expertise when performing deep chemical peels.

All patients included in this study had Fitzpatrick skin phototypes I to III. Proper patient selection is critical to minimizing pigmentary complications, particularly demarcation and depigmentation in darker skin types. For this reason, patients with Fitzpatrick phototypes IV to VI are generally not considered ideal candidates for deep peels, as they are at increased risk for adverse pigmentary outcomes and typically do not present with pronounced photoaging [9].

## 5 Strengths and Limitations

Phenol‐croton peel has lately gained popularity due to excellent outcomes reported on social media. Dermatologists are aware of side effects that may follow this procedure; however, in medical literature, data regarding percentages of cutaneous side effects expected after a phenol‐croton peel is limited, contributing to the avoidance of performing this procedure even among experienced cosmetic dermatologists. Patients are afraid of the procedure due to the downtime and uncertainty of side effects and side effect rates. Our group has set up a Dermatology outpatient clinic devoted to the treatment of skin aging using chemical peels and has gained experience in performing the procedure. Therefore, we found it appropriate to design a study to find out what cutaneous side effects and their rates would be expected following a phenol‐croton peel. We were able to reach 102 patients who had performed the procedure. Our study has shown the rates of expected short‐term and long‐term cutaneous side effects, as well as the GSI among patients, phototypes ranging from I to III, data that was missing in the literature.

This study was performed by a group of highly trained dermatologists in one single setting, following a strict protocol during patient selection and performance of the procedure. Therefore, it may not reflect the exact scenario when the procedure is performed by untrained doctors or on groups of patients with higher skin phototypes. This is a limitation of our study; new multicenter studies with larger samples may help us to confirm the rates of cutaneous side effects among other skin phototypes.

Another limitation of this study is that data on adverse effects were collected retrospectively through participant interviews. Since some of the procedures had been performed several months earlier, there is a possibility of recall bias, as participants may not have accurately remembered all the details regarding the onset, duration, or intensity of the side effects.

Although the management of adverse events during the study period could potentially introduce a confounding factor, only two patients with persistent events beyond 3 months received targeted treatments. One patient reporting prolonged skin dryness was managed conservatively with emollient moisturizers, while another with persistent hyperpigmentation was treated with a topical depigmenting agent containing hydroquinone. All other cases of persistent pigmentation changes, including both hyperpigmentation and hypopigmentation, were not subjected to any specific intervention; patients were only instructed to apply sunscreen daily. Given the limited scope and mild nature of these interventions, we believe they had minimal influence on the study's overall findings.

## 6 Conclusions

The main points of this study are that the absence of cutaneous side effects, increase in age, and full face type were predictors of better GSI among a cohort of patients who underwent phenol‐croton peel.

Phenol‐croton peels lead to a high degree of patient satisfaction even several months or years after the procedure. Cutaneous side effects should not be underestimated, as this cohort demonstrates that up to 15.68% of cutaneous side effects persist for 3 months after the procedure, even with experienced dermatologists. Fortunately, these cutaneous side effects tended to reduce over time, as observed during the second follow‐up conducted at least 15 months after the peeling procedure. This reduction was statistically significant compared to the first follow‐up. Hypochromia remained in six patients at the second follow‐up, but five of them reported they were overall satisfied and would still undergo the procedure, considering the improvement in skin rejuvenation. Methods to improve hypochromic scars have recently been investigated by our group [18], among others [19], and this promising alternative should be further investigated to treat patients with hypochromic scars after deep peels.

Photorejuvenation procedures currently include injectable products or technologies produced by multimillion industries, requiring large investments and sharing profit with dermatologists; those last ones, however, remain with all responsibility over patient satisfaction and side effects. Compounded Phenol peel formulas, on the other hand, are low cost and, as we have shown, lead to long‐term improvement and high satisfaction rates. We believe that the experience of our group might contribute to the safety guidance of younger dermatologists to perform phenol peels.

## Author Contributions

Overall study design, review, bibliographic review, ethical approval, and supervision: G.V.O. and C.G.W. Writing and literature review: M.R.A., B.A.R., A.C.T.R.M., and M.V.R.Q. Biostatistical analysis, statistical calculations, and the interpretation of collected data: M.R.S., B.A.R., and M.R.A. Study design and performance of the procedures, patient follow up and selection: G.C.N., R.I.F.M.O., N.C.N., T.T.C.C., and F.X.C. Data collection, the construction of data tables, and the application of the ICF: B.A.R. and M.R.A. Supervision and final language editing and correction: C.G.W. and G.C.N.

## Ethics Statement

The project was approved by the Ethical Committee of Faculdade de Ciências Médicas de Minas Gerais (5.943.455), Belo Horizonte and registered at Plataforma Brasil.

## Consent

Consent for the publication of recognizable patient photographs or other identifiable material was obtained by the authors and included at the time of article submission to the journal stating that all patients gave consent with the understanding that this information may be publicly available. All participants provided informed consent through an Informed Consent Form. During the initial phone call, participants were informed about the study procedures and notified that they would receive the TCLE digitally. Immediately after the call, the form was sent, and participants completed and signed it electronically before being included in the study.

## Conflicts of Interest

G.V.O. has received compensation from USK Dermatology in a project not related to this manuscript, as well as equipment from Vydence (also not related to this project). Dr. Wambier advices Young Pharmaceuticals which commercializes Novisol and is a co inventor in the USPTO #11253465 on Methods of emulsification of Phenol Croton Oil Formulas, and receives Royalties from the sales of Novisol, however those products have not been used in this study. The other authors declare no conflicts of interest. The work was conducted at the Faculty of Medical Sciences and Completa Dermatologia.

## Data Availability

The data that support the findings of this study are available on request from the corresponding author. The data are not publicly available due to privacy or ethical restrictions.
